# Editorial: Dietary bioactive compounds as coadjuvants in antiviral preventive and curative strategies

**DOI:** 10.3389/fnut.2026.1940194

**Published:** 2026-08-10

**Authors:** João C. M. Barreira, M. Beatriz P. P. Oliveira, M. A. Prieto, Cláudia S. G. P. Pereira

**Affiliations:** 1 Mountain Research Centre (CIMO), ESA, Polytechnic Institute of Bragança, Campus de Santa Apolónia, Bragança, Portugal; 2 LAQV/REQUIMTE, Department of Chemical Sciences, Faculty of Pharmacy, University of Porto, Porto, Portugal; 3 Department of Analytical Chemistry and Food Science, Instituto de Agroecoloxía e Alimentación (IAA) – CITEXVI, Universidade de Vigo, Nutrition and Bromatology Group, Vigo, Spain

**Keywords:** antiviral strategies, dietary bioactive compounds, immunomodulation, natural products, nutraceuticals

The COVID-19 pandemic profoundly reshaped our understanding of infectious diseases, emphasizing that effective antiviral preparedness requires approaches extending beyond the development of vaccines and antiviral drugs. While pharmacological therapies remain indispensable, growing evidence indicates that nutritional status and dietary bioactive compounds can significantly influence host susceptibility, immune ability, disease progression, and therapeutic results. Consequently, naturally occurring bioactive molecules are increasingly being investigated as alternatives to conventional antivirals and as complementary agents capable of enhancing antiviral efficacy, modulating excessive inflammatory responses, and improving overall host resilience.

The Research Topic *Dietary Bioactive Compounds as Coadjuvants in Antiviral Preventive and Curative Strategies*, brings together contributions illustrating the breadth of contemporary research in this rapidly evolving field. Although the published articles examine diverse classes of natural compounds, biological systems, and viral targets, they collectively converge on a central concept: successful antiviral interventions are increasingly requiring multidisciplinary approaches integrating nutrition, food science, pharmacology, virology, immunology, and medicinal chemistry.

A first major theme emerging from this Research Topic concerns the remarkable mechanistic diversity through which dietary bioactive compounds may interfere with viral infections. Unlike conventional antiviral drugs, which frequently target a single viral protein, naturally occurring compounds often exhibit pleiotropic activities, simultaneously modulating viral replication, host-cell signaling, oxidative stress, inflammation, and immune function. Such multimodal activity may be particularly valuable in addressing complex viral diseases, where both viral burden and dysregulated host responses contribute to pathology.

This concept is exemplified by the complementary reviews addressing antiviral polysaccharides. The articles on edible marine algae-derived sulfated polysaccharides and on natural and modified hydrocolloids demonstrate that these macromolecules interfere with multiple stages of the viral life cycle, including viral attachment, cell entry, intracellular replication, and immune regulation. Importantly, both reviews move beyond simply cataloging antiviral compounds by highlighting how physicochemical characteristics (including molecular weight, sulfation patterns, branching architecture, and chemical modification) are the major determinants of biological activity. Together, these contributions reinforce an increasingly important paradigm within natural-product research: antiviral efficacy depends not only on chemical composition but also on molecular structure and functionality.

The original research articles broaden this mechanistic perspective by illustrating complementary strategies for antiviral intervention. Rather than directly targeting viruses, the clinical investigation evaluating zinc deficiency in hospitalized pediatric COVID-19 patients underscores the importance of host nutritional status in determining disease severity and clinical progression. Micronutrient deficiencies may compromise immune competence and exacerbate inflammatory responses, suggesting that nutritional optimization represents an integral component of antiviral preparedness rather than a secondary consideration. In contrast, the studies investigating formononetin in combination with mizoribine against Porcine Reproductive and Respiratory Syndrome Virus and the RNA-dependent RNA polymerase inhibitory activity of *Ephedrae Herba* identify naturally derived molecules capable of directly interfering with viral replication. Although these studies focus on different viral systems and experimental models, they collectively illustrate how dietary or medicinal plant-derived compounds may complement established antiviral therapies through distinct yet potentially synergistic mechanisms.

A second major message emerging from this Research Topic concerns the increasing convergence between nutrition and pharmacology. Historically, these disciplines have largely progressed independently: nutrition focused on maintaining physiological health, whereas pharmacology intends to treat disease through highly specific molecular targets. The contributions assembled here challenge this traditional separation by demonstrating that dietary bioactive compounds frequently occupy the interface between both disciplines. Natural compounds capable of modulating immune responses, reducing oxidative stress, inhibiting viral enzymes, or potentiating existing antiviral drugs should no longer be viewed exclusively as nutritional components or pharmacological agents, but rather as multifunctional bioactive molecules whose clinical value may derive from their integration within broader therapeutic strategies.

Importantly, the Research Topic also illustrates the maturation of antiviral natural-product research. Earlier investigations often reported antiviral activity without elucidating underlying mechanisms, limiting translational potential. In contrast, the studies presented here consistently seek mechanistic understanding by identifying molecular targets, investigating structure-activity relationships, evaluating synergistic interactions, or linking nutritional biomarkers with clinical outcomes. This progression reflects an encouraging shift from descriptive observations toward evidence capable of supporting rational therapeutic development.

Despite these advances, significant challenges remain before dietary bioactive compounds can be routinely incorporated into antiviral prevention and treatment strategies. Natural products exhibit considerable variability arising from species, cultivation conditions, extraction procedures, and processing technologies, making standardization essential for reproducibility. Likewise, many promising compounds continue to face limitations related to bioavailability, pharmacokinetics, dosage optimization, and regulatory approval. Addressing these issues will require multidisciplinary collaborations involving food chemists, pharmacologists, clinicians, analytical scientists, and regulatory agencies.

Future research should also move beyond evaluating isolated compounds. Synergistic interactions among multiple bioactive molecules, dietary patterns, micronutrient status, gut microbiota, and conventional antiviral drugs represent promising paths for investigation. Advances in metabolomics, systems biology, artificial intelligence, and network pharmacology are expected to accelerate the identification of bioactive combinations capable of simultaneously targeting viral replication and host defense mechanisms. Such integrated approaches align closely with emerging concepts of precision nutrition and personalized medicine, where preventive and therapeutic interventions are increasingly tailored according to individual biological characteristics.

Collectively, the contributions gathered in this Research Topic demonstrate that dietary bioactive compounds constitute a scientifically credible and rapidly expanding field within antiviral research. Rather than promoting natural products as replacements for established antiviral therapies, the studies consistently support their role as evidence-based complementary strategies capable of enhancing host resilience, improving therapeutic efficacy, and expanding the range of antiviral interventions. As our understanding of host-virus interactions continues to evolve, integrating nutritional sciences with pharmacology and virology will become increasingly important for developing holistic and sustainable approaches to infectious disease prevention and treatment.

We hope that this Research Topic will stimulate further interdisciplinary collaborations and inspire future investigations aimed at translating promising dietary bioactive compounds into clinically validated coadjuvants that complement existing antiviral preventive and curative strategies.

